# Liver-Derived Exosomes Induce Inflammation and Lipogenesis in Mice Fed High-Energy Diets

**DOI:** 10.3390/nu14235124

**Published:** 2022-12-02

**Authors:** Jihee Lee, Inae Jeong, Ok-Kyung Kim

**Affiliations:** 1 Division of Food and Nutrition, Chonnam National University, Gwangju 61186, Republic of Korea; 2 Human Ecology Research Institute, Chonnam National University, Gwangju 61186, Republic of Korea

**Keywords:** exosome, NAFLD, lipogenesis, high-energy diets

## Abstract

The liver is an endocrine organ and is the first organ exposed to nutrients when they are absorbed into the body before being metabolized by the distal organs. Although the liver plays an essential role in the interactions between the metabolic organs, their regulatory mechanisms have not been elucidated. Exosomes mediate communication between cells and primarily enable the transport of lipids, mRNAs, miRNAs, and proteins between cells. In this study, we investigated the effects of lipid metabolism on the liver and adipose tissue between mice fed high-fat (HF) and high-fat/sucrose (HFS) diets and determined the effects of liver tissue-derived exosomes on adipocytes to understand the underlying mechanisms associated with obesity-related metabolic diseases. Normal, HF, and HFS diets were fed to the mice for 12 weeks to compare differences based on dietary patterns. We showed different lipid metabolism effects on the liver and adipose tissue between HF- and HFS-fed mice. In the liver, fibrosis, inflammation, and lipogenesis were activated at higher levels in the HFS than in the HF group, and lipolysis was activated at higher levels in the HF than in the HFS group. In adipose tissue, adipogenesis, fatty acid transport, and lipolysis were activated at higher levels in the HF than in the HFS group, and inflammation and lipogenesis were activated at higher levels in the HFS than in the HF group. This result followed a similar trend reported in 3T3-L1 cells treated with liver-derived exosomes. In addition, the TG content of the liver-derived exosomes was significantly higher, and lipid accumulation was accelerated in the HFS than in the HF group. Based on these results, continuous exposure to HF and HFS diets induces lipid accumulation mediated by liver-derived exosomes; however, there is a difference in lipid metabolism. These results contribute to the elucidation of the mechanisms of exosome function in relation to obesity-related metabolic diseases and the metabolic relationship between tissues.

## 1. Introduction

Currently, eating patterns are characterized by overeating, frequent snacking, and consumption of sucrose-containing soft drinks. In particular, sugar is consumed excessively as an additive in beverages, sweets, and instant foods. Frequent exposure to these diets increases insulin levels, and foods with a high insulin response stimulate appetite and decrease satiety [1]. In several studies, a high-energy diet is expressed as a Western or high-density-energy diet. These diets are characterized by high-sugar and high-saturated fat foods [2,3]. Sugar is consumed mainly in sweet drinks, sweetened grains, table sugar, syrups, candies, and dairy products [4]. Sugar consumption from sugar-sweetened sodas and flavored milk is particularly high among children between the ages of 2 and 11 years [5]. The sugar intake is highest during adolescence and then decreases with age. Between 75% and 80% of adolescents with obesity develop into adults with obesity, which may have a more adverse effect on morbidity and mortality than the development of obesity in adulthood [6,7]. In this study, the obesity model was induced by a high-energy diet, and 5-week-old mice were followed-up to establish a model of adolescence.

The liver and adipose tissue are major organs for lipid metabolism and play pivotal roles in various metabolic processes in the body [8,9]. Therefore, they are closely related to the diet-induced development of metabolic syndrome [10]. Obesity and nonalcoholic fatty liver disease (NAFLD) are mainly affected by the consumption of a high-energy-density diet [11,12]. NAFLD includes various liver diseases characterized by increased liver triglyceride (TG) content [13]. NAFLD ranges from simple steatosis, nonalcoholic steatohepatitis (NASH) with or without fibrosis, and cirrhosis, an irreversible condition beyond fibrosis, to end-stage liver disease, including hepatocellular carcinoma [14,15]. Despite numerous attempts, there are so far no approved drugs to treat NAFLD [16,17]. Currently, NAFLD treatment is mainly limited to diet and lifestyle changes, but its prevalence is gradually increasing [18]. The NAFLD model was constructed in various studies using a high-fat diet (HF). A previous study showed that hepatic steatosis induced by an HF diet occurred after 1–2 weeks, then showed a tendency to decrease but reappeared after 6–12 weeks [19]. The HF diet induced obesity, insulin resistance, and hepatic steatosis in rodents but did not increase the levels of inflammation and fibrosis. HF diets, including those with sugar, increased liver TG accumulation, inflammation, and fibrosis compared with the normal HF diet. In other studies, a high-fructose HF diet increased intrahepatic lipid deposition compared to the HF diet, and hepatic TG accumulation was associated with metabolic diseases, including obesity and insulin resistance [20,21,22].

Recently, exosomes have been attracting attention as cell signaling agents. Exosomes contain information (e.g., DNA, mRNA, miRNA, protein, and lipid) from the donor cells and transmit information to recipient cells. It was reported that exosomes derived from hepatitis C virus-infected liver induced inflammation and fibrosis in LX-2 cells, which are human hepatic stellate cells [23]. In addition, a metabolic study showed that exosomes released from the liver of mice fed an HF diet for 12 weeks increased adipogenic factors and inflammatory factors in adipocytes and induced lipid accumulation [24]. These findings suggest that exosomes released from the liver may not only act in that same liver but also affect other tissues and may affect the development of the liver. In particular, the metabolic analysis confirmed the role of exosomes in lipid metabolism. In our previous study, exosomes derived from adipose tissue from mice fed an HF diet promoted endoplasmic reticulum (ER) stress, the inflammatory response, insulin resistance, and lipogenesis in hepatocytes. In another study, exosomes derived from the liver tissue of mice fed an HF diet were more abundant than those in the control group, increasing the rate of lipogenesis in the adipocytes [25].

An integrated understanding of the metabolic relationship among tissues is lacking. Furthermore, the link between NAFLD and lipid accumulation in adipose tissue, a major hallmark of obesity, remains unclear. In this study, we demonstrated the differences in lipid metabolism between an HF diet and a high-fat plus high-sucrose (HFS) diet in both liver and adipose tissues and the role of liver exosomes on lipid metabolism in adipocytes.

## 2. Materials and Methods

### 2.1. HF or HFS Diet–Fed Mice

Five-week-old male C57BL6/J mice were purchased from G Bio (Gwangju, Republic of Korea) and were nurtured in a controlled environment at 22–25 °C with an alternating 12 h light and dark cycle. After 1 week of acclimation, the mice were randomly divided into three groups (*n* = 8 per group) to receive a conventional normal diet (CON, AIN-93G), HF diet (AIN-93G with 60 kcal% fat), or HFS diet (AIN-93G with 60 kcal% fat + 10% sucrose water; water and sucrose water were provided alternately once a week) for 12 weeks. The animal study protocol was approved by the Institutional Animal Care and Use Committee of Chonnam National University (CNU IACUC-YB-2021-122), and the animals were cared for in accordance with the “Guidelines for Animal Experiments” established by the university.

### 2.2. Serum Biochemical Analysis

After 12 h fasting, mice were sacrificed and blood was collected in a microtube containing heparin (Sigma-Aldrich, St. Louis, MO, USA) via the inferior vena cava. Collected blood was centrifuged at 1000× *g* and 4 °C for 10 min; the supernatant was used for the serum assay. Alanine aminotransferase (ALT; BioVision, Milpitas, CA, USA, #K752-100), aspartate aminotransferase (AST; BioVision, Milpitas, CA, USA, # K753-100), glucose (Biomax, Guri-si, Republic of Korea, #BM-GLO-100), TG (Biomax, Guri-si, Republic of Korea, # BM-TGR-100), total cholesterol (TC; Biomax, Guri-si, Republic of Korea, #BM-CHO-100), high-density lipoprotein (HDL), and low-density lipoprotein (LDL) (Biomax, Guri-si, Republic of Korea, #BM-CDL-100) were measured in the serum in accordance with the respective manufacturer manuals.

### 2.3. Histological Observation

Mouse hepatic and adipose tissues were fixed overnight in a 10% neutral-buffered formaldehyde solution and then rinsed with phosphate-buffered saline. Tissues were embedded in paraffin and stained with hematoxylin–eosin (H&E) and Sirius red. Sections were then observed under a light microscope.

### 2.4. Isolation and Quantification of Liver-Derived Exosoms

The liver from mice was excised and cut into pieces (2 mm × 2 mm), and the shredded tissue was cultured in Dulbecco’s modified Eagle medium containing 10% exo-free fetal bovine serum. The exosome isolation from the culture supernatant was performed according to previously described methods [25].

### 2.5. Cell Culture

3T3-L1 cells (ATCC, Manassas, VA, USA) were cultured and differentiated according to previously described methods [26]. Differentiated 3T3-L1 cells were treated with liver-derived exosomes for 48 h.

### 2.6. Exosome Uptake Assay

The isolated exosomes were labeled using an ExoGlow-Protein EV Labeling Kit (System Biosciences, Palo Alto, CA, USA). Differentiated 3T3-L1 cells were supplemented with labeled exosomes for 48 h, and exosome uptake was confirmed according to previously described methods [25].

### 2.7. Measurement of TG Content

TG content was measured according to previously described methods [25].

### 2.8. Western Blotting

Proteins were extracted from exosomes, liver tissue, and adipose tissue and analyzed for the expression of CD63, phosphorylated hormone-sensitive lipase (p-HSL), hormone-sensitive lipase (HSL), adipocyte fatty acid-binding protein 4 (FABP4), acetyl-CoA carboxylase 1 (ACC1), phosphorylated acetyl-CoA carboxylase 1 (p-ACC1), fatty acid synthase (FAS), and beta-actin (β-actin), according to methods described previously [25].

### 2.9. RNA Isolation and Quantitative Real-Time PCR (q-PCR)

Total RNA was extracted from hepatic tissue, adipose tissue, and 3T3-L1 cells and analyzed for the expression of genes by real-time PCR using the custom-designed primers (Table 1), according to methods described previously [25].

### 2.10. Statistical Analysis

All data are presented as the mean ± standard deviation (SD). Statistical analyses were performed using a one-way Duncan’s multiple range test, followed by one-way analysis of variance (ANOVA) or Student’s *t*-test for two-sample comparison using the SPSS statistics software (SPSS PASW Statistic 23.0, SPSS, Inc., Chicago, IL, USA). Statistical significance was set at *p* < 0.05.

## 3. Results

### 3.1. Hematological and Histological Changes in HF or HFS Diet–Fed Mice

At a 12-week follow-up, the weight gain, total calorie intake, food efficiency ratio (FER), and liver and white adipose tissue weights had increased in the HF and HFS groups compared to those in the CON group. Interestingly, weight gain, FER, and weights of liver and subcutaneous white adipose tissue in the HF group were significantly greater than those in the HFS group, whereas total calorie intake had no significant difference between the HF group and the HFS group (Table 2; *p* < 0.05).

The serum levels of TG, TC, LDL, ALT, and glucose were significantly increased in the HF and HFS groups compared with those in the CON group. When comparing the HF and HFS groups, the serum levels of TG, AST, and glucose in the HFS group were significantly higher than in the HF group, whereas the serum levels of TC and LDL in the HF group were significantly higher than in the HFS group (*p* < 0.05; Table 3).

**Figure 1:**
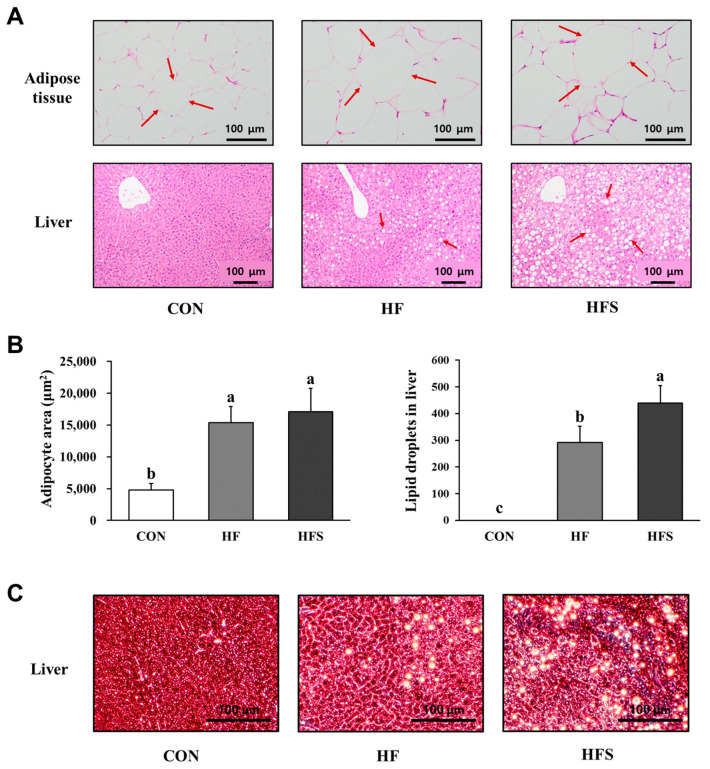
Representative images of lipid droplets observations using hematoxylin-eosin in the epididymal white adipose tissue and liver (**A**), adipocytes area in the epididymal white adipose tissue, lipid droplets in the liver (**B**), and representative images of Sirius red staining in the liver (**C**) from mice. C57BL/6J mice were fed an AIN-93G (CON), high-fat diet (HF; AIN-93G with 60 kcal% fat), or high-fat diet with 10% sucrose water (HFS) diet for 12 weeks. All data are expressed as the mean ± standard deviation. Different letters (a > b > c) indicate a significant difference among treatments. Multiple comparisons of means were performed using Duncan’s multiple range test at the 0.05 significance level (*n* = 8).

To confirm the lipid accumulation in the liver and adipose tissue, H&E staining was performed. The HF and HFS groups exhibited larger lipid droplets in both the liver and adipose tissues than the CON group. However, the contents of lipid droplets in the liver were higher in the HFS group than in the HF group, whereas there was no difference in adipose tissue lipid droplets and adipocyte area between the HF and HFS groups (*p* < 0.05; Figure 1A,B). In addition, we found that the HF and HFS groups exhibited hepatic fibrosis, especially in HFS groups, in the evaluation of collagen fiber levels using Sirius red staining (Figure 1C).

### 3.2. Lipid Metabolism-Related Factors in the Liver of HF or HFS Diet-Fed Mice

**Figure 2:**
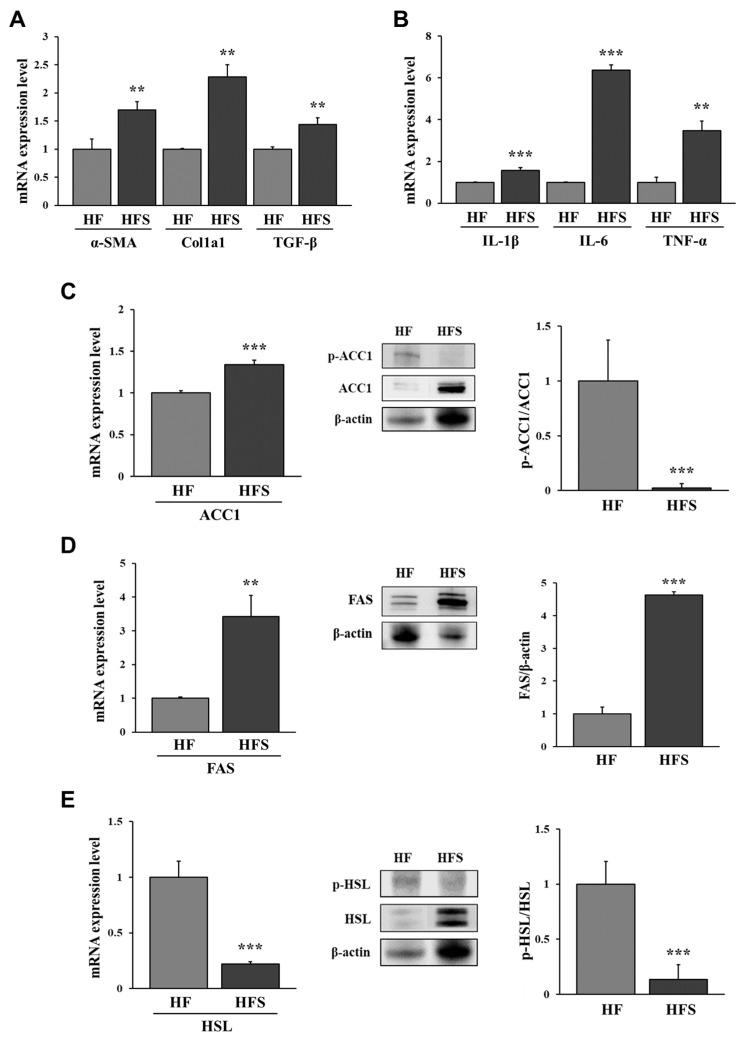
Relative mRNA expression of fibrotic factors α-SMA, Col1a1, and TGF-β (**A**) and pro-inflammatory cytokines IL-1β, IL-6, and TNF-α (**B**) and relative mRNA and protein expression of ACC1 (**C**), FAS (**D**), and HSL (**E**) in the livers of mice. C57BL/6J mice were fed a high-fat diet (HF; AIN-93G with 60 kcal% fat) or a high-fat diet with 10% sucrose water (HFS) for 12 weeks. All data are expressed as the mean ± standard deviation. Statistical analyses were performed using Student’s *t*-test (*n* = 3, HF vs. HFS; **, *p* < 0.01, ***, *p* < 0.001). α-SMA, alpha-smooth muscle actin; Col1a1, collagen type 1 alpha 1; TGF-β, transforming growth factor-beta; IL-1β, interleukin-1 beta; IL-6, interleukin-6; TNF-α, tumor necrosis factor-alpha; ACC1, acetyl-CoA carboxylase 1; FAS, fatty acid synthase; HSL, hormone-sensitive lipase.

To confirm the level of liver fibrosis, we measured the mRNA expression of fibrotic factors, including α-smooth muscle actin (α-SMA), collagen type 1 alpha 1 (Col1a1), and transforming growth factor-beta (TGF-β), in the livers of mice. The mRNA expression of α-SMA, Col1a1, and TGF-β was significantly higher in the HFS group than in the HF group (*p* < 0.05; Figure 2A). These results suggest that mild fibrosis was more likely to be induced by the HFS diet than by the HF diet.

Previous studies have reported that liver inflammation is associated with obesity-induced liver disease; thus, we measured the mRNA expression of pro-inflammatory cytokines in the liver. The HFS diet increased the mRNA expression of pro-inflammatory cytokines interleukin-1 beta (IL-1β), interleukin-6 (IL-6), and tumor necrosis factor-alpha (TNF-α) in the liver more than the HF diet (*p* < 0.05; Figure 2B). These results suggest that mild NASH was induced more often with the HFS diet than with the HF diet.

Lipid metabolism in the liver and similarly in the adipose tissue contributes to metabolic homeostasis, but an abnormal increase in liver lipogenesis can induce fatty liver disease. We compared the mRNA expression of lipogenic enzymes, including ACC1 and FAS, in the liver and found that the activation (dephosphorylation) of ACC1 significantly increased in the HFS group compared with that in the HF group (Figure 2C). In addition, the expression of FAS was significantly increased in the HFS group compared with that in the HF group (Figure 2D). We compared the expression of HSL to confirm lipolysis. The mRNA expression of HSL and phosphorylation of HSL were significantly lower in the HFS group than in the HF group (*p* < 0.05; Figure 2E). Thus, these results suggest that the HFS diet induced lipid accumulation in the liver via the activation of lipogenesis compared to the HF diet.

### 3.3. Lipid Metabolism-Related Factors in the Adipose Tissue of HF or HFS Diet-Fed Mice

**Figure 3:**
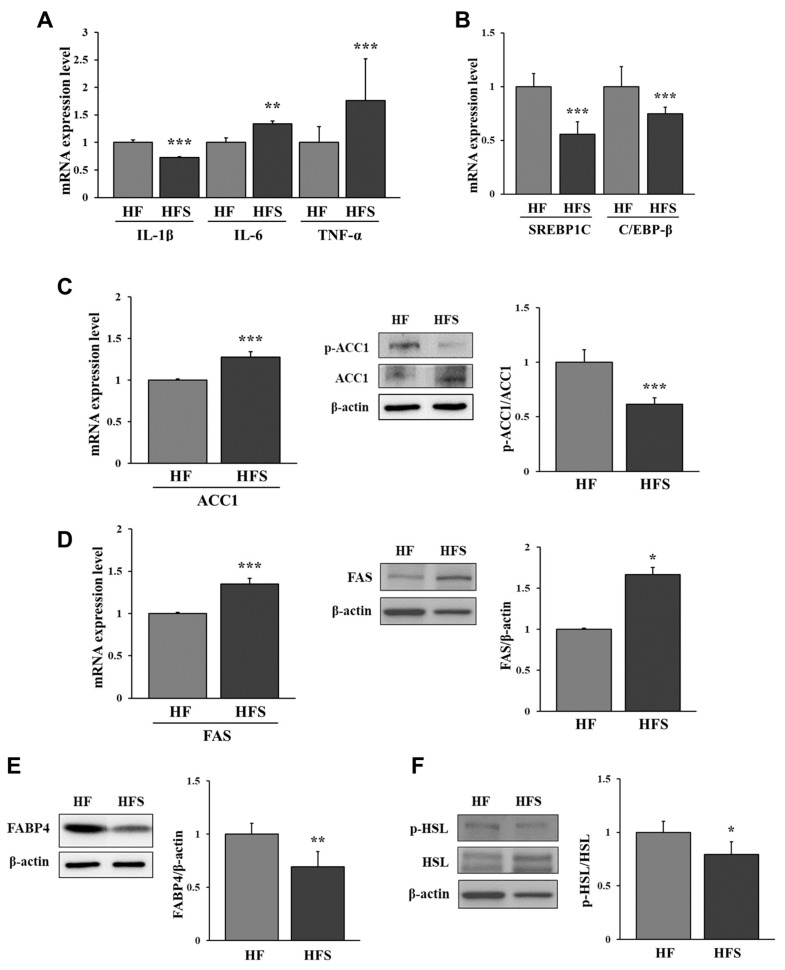
Relative mRNA expression of pro-inflammatory cytokines, IL-1β, IL-6, and TNF-α (**A**), SREBP1C, and C/EBP-β (**B**), relative mRNA and protein expression of ACC1 (**C**) and FAS (**D**), and protein expression of FABP4 (**E**) and HSL (**F**) in the epididymal white adipose tissue of mice. C57BL/6J mice were fed a high-fat diet (HF; AIN-93G with 60 kcal% fat) or a high-fat diet with 10% sucrose water (HFS) for 12 weeks. All data are expressed as the mean ± standard deviation. Statistical analyses were performed using Student’s *t*-test (*n* = 3, HF vs. HFS; *, *p* < 0.05, **, *p* < 0.01, ***, *p* < 0.001). IL-1β, interleukin-1 beta; IL-6, interleukin-6; TNF-α, tumor necrosis factor-alpha; SREBP1C, sterol regulatory element-binding protein-1C; C/EBP-β, CCAAT/enhancer-binding protein-beta; ACC1, acetyl-CoA carboxylase 1; FAS, fatty acid synthase; FABP4, fatty acid-binding protein 4, HSL, hormone-sensitive lipase.

We found that the mRNA expression of IL-6 and TNF-α in the adipose tissue of the HFS group was significantly higher than that observed in the HF group, but the mRNA expression of IL-1β in the adipose tissue from the HF group was significantly higher than that from the HFS group (*p* < 0.05; Figure 3A).

To investigate the mechanism of lipid accumulation in adipose tissue in the HF and HFS groups, lipogenic factors were identified. Surprisingly, the mRNA expression of sterol regulatory element-binding protein-1C (SREBP1C) and CCAAT/enhancer-binding protein-beta (C/EBP-β) was significantly increased in the HF group compared with that in the HFS group (Figure 3B). We found that the activation (dephosphorylation) of ACC1 was significantly increased in the HFS group compared with that in the HF group (Figure 3C). In addition, the protein and mRNA expression of FAS were significantly increased in the HFS group compared with that in the HF group (*p* < 0.05; Figure 3D). These data indicate that HFS induced lipid accumulation in the adipose tissue via activation of lipogenesis. In addition, the mRNA and protein expression of FABP4, a fatty acid transporter, was significantly increased in the HF group compared to that in the HFS group (*p* < 0.05; Figure 3E). These results indicate that adipogenesis and fatty acid transport were induced at higher levels in adipose tissue from mice that received the HF diet than those that received the HFS diet.

We compared the expression of HSL in the adipose tissue to confirm lipolysis. The mRNA expression and phosphorylation of HSL were significantly higher in the HF group than in the HFS group (*p* < 0.05; Figure 3F). Thus, these results suggest that lipolysis was activated in adipose tissue at higher levels in the HF group than in the HFS group.

### 3.4. Uptake of Liver-Derived Exosomes by Adipocytes

**Figure 4:**
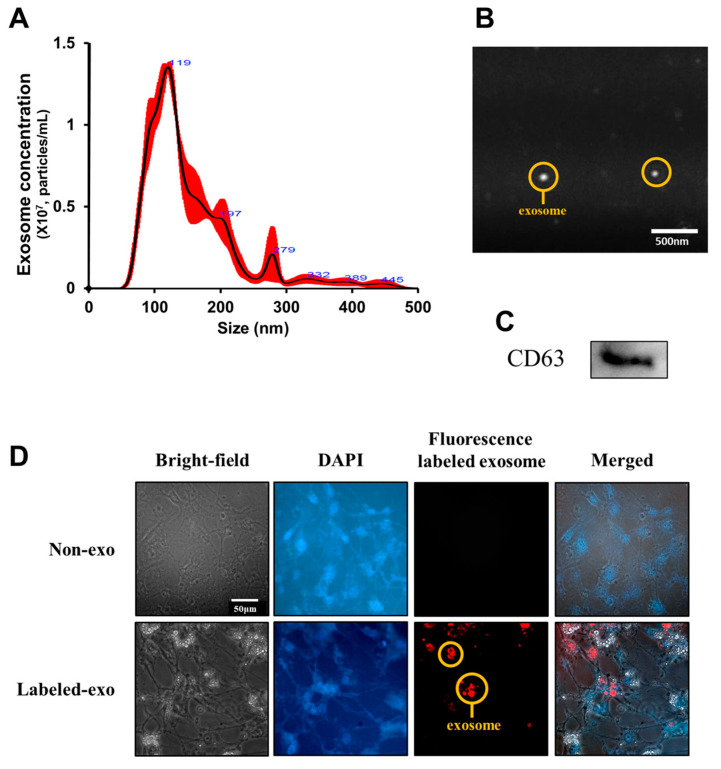
Identification and quantification of secreted liver-derived exosomes. Size distribution (**A**), images for isolated liver-derived exosomes (Scale bar = 500 nm) (**B**), expression of the exosome marker CD63 (**C**). Red fluorescence-labeled liver-derived exosome uptake in 3T3-L1 cells. Representative fluorescence microscope images show the distribution of liver-derived exosomes, as indicated by the PKH-26 red fluorescent dye, whereas cell nuclei were stained with DAPI (blue) (Scale bar = 50 μm) (**D**).

Exosomes were isolated from the liver tissue, the vesicle size was measured, and the expression of the exosome marker CD63 was determined. We found a high peak value between 50–120 nm, corresponding to the size of conventional exosomes (Figure 4). In addition, the expression of CD63 was confirmed in exosomes isolated from adipose tissue (Figure 4). Thus, we confirmed that the vesicles isolated from the liver were exosomes.

To exert a physiological effect, liver-derived exosomes must be taken up by the recipient cells. Therefore, we tagged liver-derived exosomes with a PKH-26 red fluorescent dye and then added them to the culture medium of the 3T3-L1 cells to observe the extent of exosome uptake. After 48 h of treatment, fluorescence was observed in 3T3-L1 cells (Figure 4). Thus, our results confirmed that liver-derived exosomes could be taken up by 3T3-L1 cells, suggesting a potential role of exosomes in the induction of specific physiological changes within the cells.

### 3.5. Amounts and TG Content of Liver-Derived Exosomes

**Figure 5:**
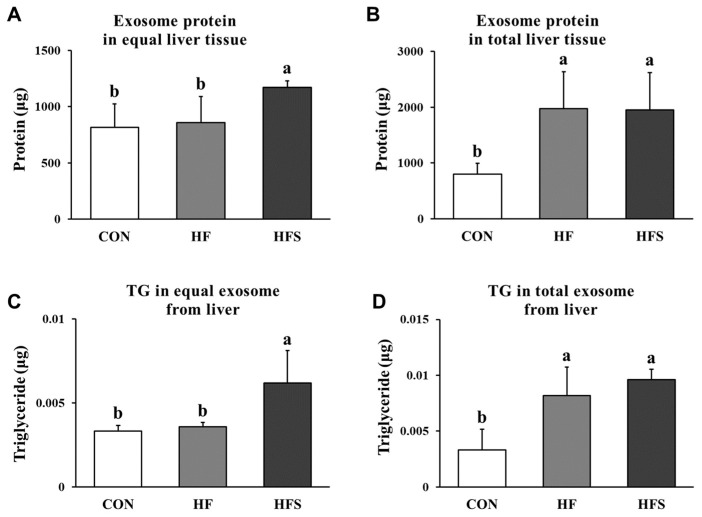
Protein content of liver-derived exosomes in livers of equal weight (**A**) and total liver weight (**B**) for mice and TG levels of liver-derived exosomes in equal amounts of exosomes (**C**) and total exosomes (**D**). C57BL/6J mice were fed an AIN-93G (CON), high-fat (HF; AIN-93G with 60 kcal% fat), or high-fat diet with 10% sucrose water (HFS) for 12 weeks. All data are expressed as the mean ± standard deviation. Different letters (a > b) indicate a significant difference among treatments. Multiple comparisons of means were performed using Duncan’s multiple range test at the 0.05 significance level (*n* = 8). TG, triglyceride.

To quantify the number of liver-derived exosomes, the total protein level was evaluated after lysing the isolated exosomes. We found that the amount of liver-derived exosomal protein for the same liver tissue weight was significantly increased in the HFS group compared with that in the CON and HF groups (Figure 5A). When comparing the amount of liver-derived exosomal protein after standardization using total liver tissue weight (Table 2), total exosome secretion from the liver was significantly increased in the HF and HFS groups compared with that in the CON group (*p* < 0.05), and there was no significant difference between the HF and HFS groups in terms of total exosome secretion from the liver (Figure 5B). We measured the TG content of the liver-derived exosomes to determine whether dietary intake affects this amount. The TG content of equal amounts of liver-derived exosomes was significantly increased in the HFS group compared with that in the CON and HF groups (Figure 5C). The TG content of the total exosomes from total liver tissue was significantly increased in the HF and HFS groups compared with that in the CON group (*p* < 0.05), and there was no significant difference between the HF and HFS groups (Figure 5D). These results suggest that 12 weeks of HFS feeding increased the TG contents of liver-derived exosomes.

### 3.6. TG Levels in Adipocytes Treated with Liver-Derived Exosomes

**Figure 6:**
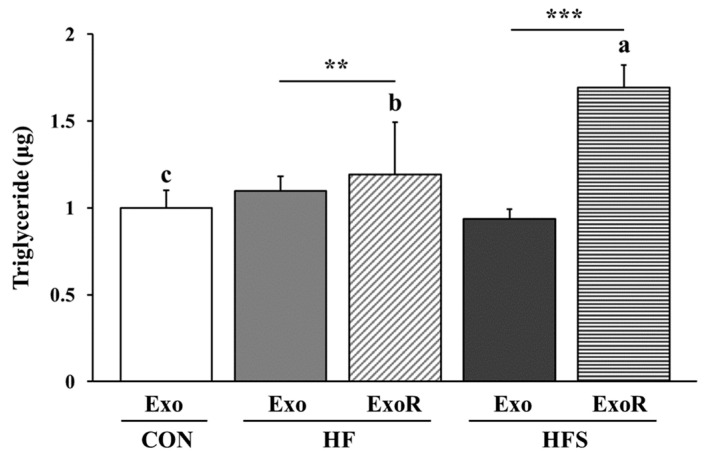
Triglyceride levels in differentiated 3T3-L1 cells treated with liver-derived exosomes from mice. C57BL/6J mice were fed an AIN-93G (CON), high-fat (HF; AIN-93G with 60 kcal% fat), or high-fat diet with 10% sucrose water (HFS) for 12 weeks. Cells were treated with 50 µg/mL of liver-derived exosomes from the CON (CON-Exo), 50 µg/mL exosomes from the HF (HF-Exo), 50 µg/mL exosomes from the HFS (HFS-Exo), 122.5 µg/mL exosomes from the HF (HF-ExoR), and 121.5 µg/mL exosomes from the HFS (HFS-ExoR) groups. The levels of HF-ExoR and HFS-ExoR were obtained from the differential ratio to normalize the values to the weight of liver tissue. All data are expressed as the mean ± standard deviation. Different letters (a > b > c) indicate significant differences among CON-Exo, HF-ExoR, and HFS-ExoR. Multiple comparisons of means were performed using Duncan’s multiple range test at the 0.05 significance level. Statistical analyses were performed using Student’s *t*-test (*n* = 3, Exo vs. ExoR; **, *p* < 0.01, ***, *p* < 0.001).

We evaluated the TG levels in differentiated 3T3-L1 cells after treatment with liver-derived exosomes to determine whether these exosomes affected lipid accumulation in adipocytes. Cells were treated with 50 µg/mL of liver-derived exosomes from the CON (CON-Exo), 50 µg/mL exosomes from the HF (HF-Exo), 50 µg/mL exosomes from the HFS (HFS-Exo), 122.5 µg/mL exosomes from the HF (HF-ExoR), and 121.5 µg/mL exosomes from the HFS (HFS-ExoR) groups. The levels for HF-ExoR and HFS-ExoR were obtained from the differential ratio to normalize the values to the weight of liver tissue. We found that the TG level was significantly increased in the differentiated 3T3-L1 cells treated with HFS-ExoR compared to those treated with HF-ExoR (*p* < 0.05; Figure 6). This result suggests that the liver-derived exosomes from the HFS group had a greater effect on lipid accumulation in adipocytes compared to the other groups.

### 3.7. Lipid Metabolism-Related Factors in Adipocytes Treated with Liver-Derived Exosomes

**Figure 7:**
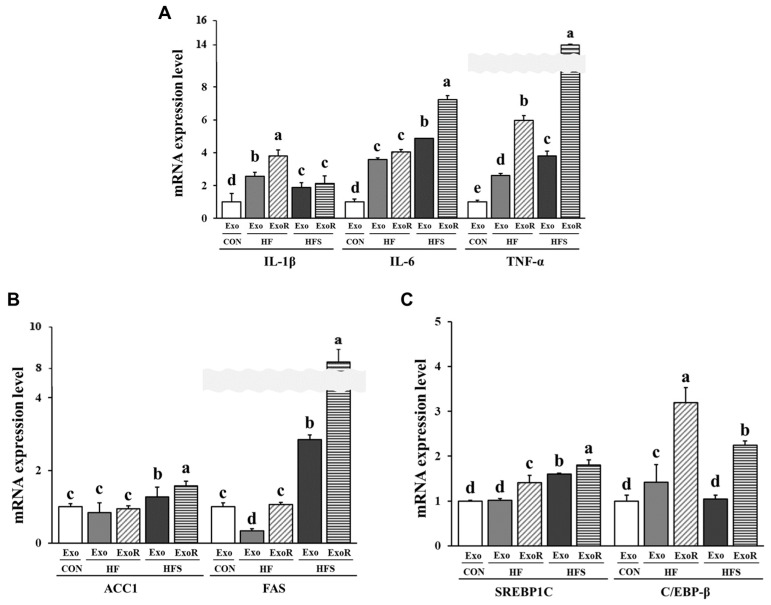
Relative mRNA expression of pro-inflammatory cytokines, IL-1β, IL-6, and TNF-α (**A**), ACC1, FAS (**B**), SREBP1C, and C/EBP-β (**C**) in differentiated 3T3-L1 cells treated with liver-derived exosomes for 48 h. C57BL/6J mice were fed an AIN-93G (CON), high-fat (HF; AIN-93G with 60 kcal% fat), or high-fat diet with 10% sucrose water (HFS) for 12 weeks. Cells were treated with 50 µg/mL of liver-derived exosomes from the CON (CON-Exo), 50 µg/mL exosomes from the HF (HF-Exo), 50 µg/mL exosomes from the HFS (HFS-Exo), 122.5 µg/mL exosomes from the HF (HF-ExoR), and 121.5 µg/mL exosomes from the HFS (HFS-ExoR) groups. The amounts of HF-ExoR and HFS-ExoR were obtained from the differential ratio to normalize the values to the weight of liver tissue. All data are expressed as the mean ± standard deviation. Different letters (a > b > c > d > e) indicate a significant difference among treatments. Multiple comparisons of means were performed using Duncan’s multiple range test at the 0.05 significance level (*n* = 3). IL-1β, interleukin-1 beta; IL-6, interleukin-6; TNF-α, tumor necrosis factor-alpha; ACC1, acetyl-CoA carboxylase 1; FAS, fatty acid synthase; SREBP1C, sterol regulatory element-binding protein-1C; C/EBP-β, CCAAT/enhancer-binding protein-beta.

To investigate the mechanism of lipid accumulation in adipocytes, we confirmed the inflammatory response and lipogenic and adipogenic factors of liver-derived exosomes in 3T3-L1 cells after treatment. The mRNA expression of IL-1β was significantly increased in the differentiated 3T3-L1 cells treated with HF-ExoR compared to that in the other cells. The mRNA expression of IL-6 and TNF was significantly increased in the differentiated 3T3-L1 cells treated with HFS-ExoR compared to that in the other cells (*p* < 0.05; Figure 7A). This result suggests that the liver-derived exosomes could affect the development of adipocyte inflammation.

We investigated whether liver-derived exosomes regulate the processes of lipogenesis and adipogenesis in differentiated 3T3-L1 cells. The mRNA expression of lipogenic enzymes ACC1 and FAS (Figure 7B) was significantly increased in the differentiated 3T3-L1 cells treated with HFS-ExoR compared to that in the other cells. However, the mRNA expression of C/EBP-β was significantly increased in the differentiated 3T3-L1 cells treated with HF-ExoR compared to that in the other cells, but mRNA expression of SREBP1C was significantly increased in the differentiated 3T3-L1 cells treated with HFS-ExoR compared to that in the other cells (*p* < 0.05; Figure 7C).

## 4. Discussion

The incidence of NAFLD and obesity and their associated complications are on the rise. NAFLD is closely related to obesity, and although the relationship between obesity and NAFLD is still unknown, NAFLD occurs as a complication of obesity [27]. The development of obesity is associated with the classic Western diet, which is known for its high saturated fat and sugar contents [28]. This eating pattern, in combination with NAFLD and obesity, causes the occurrence of dyslipidemia, hyperglycemia, adipose tissue hypertrophy, and lipid droplet accumulation in liver tissue [29,30].

Recent studies reported the pathological function of adipose tissue-derived exosomes in the pathogenesis of various diseases [31,32,33]. The composition of substances contained in exosomes varies depending on the state of the donor cells [34]. In a previous study, we found that adipose tissue-derived exosomes released from obese mice fed an HF diet induced ER stress, insulin resistance, lipogenesis, and inflammation in AML12 cells (murine hepatocytes). In addition, adipose tissue-derived exosomes released from obese mice induced insulin resistance and TG accumulation in C2C12 cells (murine muscle cells) [25]. Therefore, in the present study, the effect of exosomes released from the liver, which is closely related to nutrient metabolism, on adipose tissue and adipocytes based on the eating pattern of mice was investigated. Normal, HF, and HFS diets were fed to mice to compare the differences in metabolic processes according to the dietary patterns. In addition, obesity was induced, with a follow-up period of 12 weeks to examine changes through long-term exposure to the diets.

Interestingly, although body weight, liver weight, and LDL-cholesterol in the HF group increased more than in the HFS group, the serum levels of TG, AST, and glucose and lipid accumulation in the liver showed a higher trend in the HFS group than in the HF group. These results indicate that sucrose increased blood TG levels and glucose concentrations, stimulated liver damage to increase AST levels and that cholesterol metabolism was significantly increased in the HF group than in the HFS group. Thus, we predict that very-low-density lipoprotein release from the liver may have been inhibited by the HFS diet, resulting in increased lipid accumulation in the liver.

To demonstrate the differences in lipid metabolism in the liver between the HF and HFS groups, factors associated with fibrosis, inflammation, lipogenesis, and lipolysis in the liver were measured. Interestingly, a clear difference between the HF and HFS group was confirmed for most factors. Fibrosis, inflammation, and lipogenesis were activated in the HFS group compared to those in the HF group, and lipolysis was activated at higher levels in the liver in the HF group than in the HFS group. Therefore, the increase in lipid accumulation in the liver of the HFS group, as confirmed through H&E staining, can be interpreted as a result of the activation of lipogenesis. We suggest that the HFS-induced lipid accumulation in the liver could induce hepatic damage and accelerate fibrosis.

H&E staining of the adipose tissue confirmed no difference in the size of lipid droplets between the HF and HFS groups. However, we identified differences in lipid metabolism between the HF and HFS groups in the liver and adipose tissue. We showed that adipogenesis, fatty acid transport, and lipolysis were activated at higher levels in the HF than in the HFS group, and inflammation and lipogenesis were activated at higher levels in the HFS group than in the HF group in the adipose tissue. These results indicate that the HFS group displayed no difference in lipid droplet size because lipogenesis was activated at higher levels, but lipolysis was inhibited in this group compared to that in the HF group.

In recent studies, the secretion of exosomes was increased in the liver of obese mice [35,36]. In the present study, we compared liver-derived exosomes among the CON, HF, and HFS groups using liver tissues of equal weight and found that the liver-derived exosomal protein amount in the HFS group was higher than that in the CON and HF groups. However, because the total liver tissue weights differed for each group, the number of exosomes secreted per animal was calculated considering the total liver tissue weight. The number of exosomes from whole liver tissue can be considered as circulating liver-derived exosomes in the blood of mice. We showed that the number of exosomes from whole liver tissue increased in the HF and HFS groups compared with that in the CON group, and there was no significant difference between the HF and HFS groups. These results indicate that HFS induced the secretion of liver-derived exosomes, but there was no difference between the HF and HFS groups in terms of the amount of circulating liver-derived exosomes when considering the liver weight.

In addition, we predicted that the TGs would be internally released in exosomes derived from liver tissue; thus, the TG levels in exosomes were measured. There was no difference in the TG levels of the total liver exosomes between the HF and HFS groups, but TG levels in equal amounts of liver-derived exosomes were approximately doubled in the HFS group than in the HF group. These results suggest that the levels of exosome secretion and endogenous TGs are increased by the HFS diet, which may affect cell communication.

To demonstrate the link between NAFLD and lipid accumulation in adipose tissue, we treated the adipocytes with the isolated liver-derived exosomes and measured the TG accumulation and expression of factors associated with inflammation, lipogenesis, and adipogenesis in adipocytes. When considering the amount of circulating liver-derived exosomes, TG accumulation was found to be increased in 3T3-L1 cells treated with liver-derived exosomes from the HFS group, and the expression of lipogenic and inflammatory factors was higher in the HFS-ExoR treatment group than in the HF-ExoR treatment group. However, the expression of adipogenic factors was higher in the HF-ExoR treatment group than in the HFS-ExoR treatment group. These findings suggest that, in an obese state, liver-derived exosomes further exacerbate obesity and that the sucrose diet accelerates the effect of the liver-derived exosomes. Furthermore, the results suggest that liver-derived exosomes can induce chronic obese conditions in adipocytes and play the role of a risk factor for metabolic syndrome when the HFS diet is followed rather than the HF diet alone.

In this study, obesity was induced in mice through HF and HFS diets, and the effect of liver tissue-derived exosomes on 3T3-L1 cells was confirmed. However, the activation of enzymes contributing to lipogenesis and lipolysis in the liver tissue-derived exosome-treated 3T3-L1 cells requires further confirmation. The correlation between the various effects of liver-derived exosomes in other tissues is unclear. Additional studies are needed to determine the key contents and molecules of liver-derived exosomes associated with each diet and to elucidate the molecular mechanism of the effect of liver-derived exosomes in adipose tissue.

## 5. Conclusions

Our findings revealed the differences in the effects of the different lipid metabolic processes on the liver and adipose tissues between mice fed HF and HFS diets and determined the effect of liver tissue-derived exosomes on 3T3-L1 cells. In the liver, fibrosis, inflammation, and lipogenesis were activated at higher levels in the HFS group than in the HF group, and lipolysis was activated in the liver at higher levels in the HF group than in the HFS group. In the adipose tissue, adipogenesis, fatty acid transport, and lipolysis were activated at higher levels in the HF group than in the HFS group, and inflammation and lipogenesis were activated at higher levels in the HFS group than in the HF group. These results confirmed a similar trend in 3T3-L1 cells treated with liver-derived exosomes. In addition, the TGs inherent to the liver-derived exosomes were present at significantly higher levels, and lipid accumulation was accelerated in the HFS group than in the HF group. Based on these results, it was confirmed that continuous exposure to HF and HFS diets induces lipid accumulation mediated by liver-derived exosomes, but there was a difference in lipid metabolism between the two groups. Therefore, the results of this study contribute to the elucidation of the underlying mechanisms of exosome function in relation to obesity-related metabolic diseases and metabolic relationships between tissues.

## Figures and Tables

**Table 1:** Primer sequences used in real-time PCR quantification of mRNA.

Gene	Primer Sequences
*ACC1*	F: 5′-GATGAACCATCTCCGTTG-3′
	R: 5′-CCCAATTATGAATCGGGA-3′
*FAS*	F: 5′-ACTGCCTTCGGTTCAGTCTC-3′
	R: 5′-CACCCTCCAAGGAGTCTCAC-3′
*SREBP1C*	F: 5′-TGAAGACAGATGCAGGAG-3′
	R: 5′-ATGGTCCCTCCACTCACC-3′
*C/EBP-β*	F: 5′-GACAAGCTGAGCGACGAG-3′
	R: 5′-GTCAGCTCCAGCACCTTG-3′
*IL-1β*	F: 5′-GCCACCTTTTGACAGTGATG-3′
	R: 5′-ATCAGGACAGCCCAGGTCAA-3′
*IL-6*	F: 5′-CCAAGAGATAAGCTGGAGTCA-3′
	R: 5′-GCACTAGGTTTGCCGAGTAGA-3′
*TNF-α*	F: 5′ AAGTTCCCAAATGGCCTCCC 3′
	R: 5′-TTTGCTACGACGTGGGCTAC-3′
*α-SMA*	F: 5′-TCACCATTGGAAACGAACGC-3′
	R: 5′-GCTGTTATAGGTGGTTTCGT-3′
*Col1a1*	F: 5′-AGCACGTCTGGTTTGGAGAG-3′
	R: 5′-GACATTAGGCGCAGGAAGGT-3′
*TGF-β*	F: 5′-CATCCATGACATGAACCGGC-3′
	R: 5′-GTTGGTATCCAGGGCTCTCC-3′
*ACS1*	F: 5′-CCGCGACTCCTTAAATAGCA-3′
	R: 5′-GGGTTGGTGGTTCTCTATGC-3′
*HSL*	F: 5′-GTGAATGAGATGGCGAGGGT-3′
	R: 5′-GTGCCCTCACAGCAGGAATA-3′
*FABP4*	F: 5′-TGGGATGGAAAGTCGACCAC-3′
	R: 5′-TTCTTTGGCTCATGCCCTT-3′
*GAPDH*	F: 5′-AACTTGGCATTGTGGAAGG-3′
	R: 5′-CACATTGGGGGTAGGAACAC-3′

**Table 2:** Changes in BW, diet intake, FER, and organ weight from mice fed a conventional normal, HF, and HFS diet.

		CON (*n* = 8)	HF (*n* = 8)	HFS (*n* = 8)
BW (g)	Initial (0 weeks)	20.88 ± 0.6 ^NS^	20.46 ± 0.9	20.14 ± 0.7
	Final (12 weeks)	32.16 ± 2.0 ^c^	46.30 ± 2.7 ^a^	41.65 ± 4.1 ^b^
Weight gain (g)		11.27 ± 2.4 ^c^	25.83 ± 2.5 ^a^	21.51 ± 3.8 ^b^
Diet intake (g)		239.91 ± 3.1 ^NS^	230.56 ± 2.6	218.76 ± 3.3
Sucrose water intake (mL)		-	-	251.69 ± 4.9
Total calorie intake (kcal) ^1^		904.4 ± 44.58 ^b^	1164.9 ± 48.63 ^a^	1154.8 ± 60.99 ^a^
FER ^2^		1.24 ± 0.06 ^a^	2.22 ± 0.09 ^c^	1.87 ± 0.1 ^b^
Liver (g)		1.01 ± 0.1 ^c^	2.09 ± 0.3 ^a^	1.59 ± 0.4 ^b^
Total WAT (g)		2.94 ± 0.8 ^b^	6.74 ± 1.4 ^a^	6.02 ± 0.8 ^a^
Epididymal WAT (g)		1.12 ± 0.2 ^b^	2.03 ± 0.3 ^a^	2.15 ± 0.2 ^a^
Visceral WAT (g)		0.52 ± 0.2 ^b^	1.14 ± 0.3 ^a^	1.00 ± 0.2 ^a^
Subcutaneous WAT (g)		1.29 ± 0.3 ^c^	3.56 ± 0.7 ^a^	2.88 ± 0.3 ^b^
Liver weight/BW (%)		3.18 ± 0.2 ^b^	4.5 ± 0.6 ^a^	3.78 ± 0.9 ^b^
Total WAT weight/BW (%)		9.06 ± 1.9 ^b^	14.62 ± 2.6 ^a^	14.56 ± 1.1 ^a^

^1^ Total calorie intake = Calorie of each diet (kcal/g) or sucrose water (kcal/mL) × intake (g); ^2^ FER = body weight gain (g)/total calorie intake (kcal)) × 100). C57BL/6J mice were fed an AIN-93G diet (CON), a high-fat diet (HF; AIN-93G with 60 kcal% fat), or a high-fat diet with 10% sucrose water (HFS) for 12 weeks. All data are expressed as the mean ± standard deviation. Different superscript letters (a > b > c) indicate a significant difference among treatments. Multiple comparisons of means were performed using Duncan’s multiple range test at the 0.05 significance level (*n* = 8). BW, body weight; NS, not significant; FER, food efficiency ratio; WAT, white adipose tissue.

**Table 3:** Levels of serum triglyceride, total cholesterol, LDL, HDL, glucose, AST, and ALT in mice fed a conventional normal, HF, and HFS diet.

	CON (*n* = 8)	HF (*n* = 8)	HFS (*n* = 8)
Triglyceride (nmol/μL)	6.52 ± 0.30 ^c^	8.39 ± 0.45 ^b^	10.35 ± 0.32 ^a^
Total cholesterol (μg/μL)	82.97 ± 4.9 ^c^	109.98 ± 4.71 ^a^	98.83 ± 6.04 ^b^
LDL (μg/μL)	0.15 ± 0.01 ^c^	0.32 ± 0.01 ^a^	0.23 ± 0.01 ^b^
HDL (μg/μL)	0.12 ± 0.02 ^b^	0.27 ± 0.02 ^a^	0.26 ± 0.02 ^a^
Glucose (nmol/μL)	1.8 ± 0.08 ^c^	2.9 ± 0.1 ^b^	3.5 ± 0.02 ^a^
AST (U/mL)	2.9 ± 1.0 ^c^	4.3 ± 1.1 ^ab^	5.4 ± 1.5 ^a^
ALT (mU/mL)	0.46 ± 0.05 ^b^	1.7 ± 0.11 ^a^	1.59 ± 0.05 ^a^

C57BL/6J mice were fed an AIN-93G diet (CON), a high-fat diet (HF; AIN-93G with 60 kcal% fat), or a high-fat diet with 10% sucrose water (HFS) for 12 weeks. All data are expressed as the mean ± standard deviation. Different superscript letters (a > b > c) indicate a significant difference among treatments. Multiple comparisons of means were performed using Duncan’s multiple range test at the 0.05 significance level (*n* = 8). LDL, low-density lipoprotein; HDL, high-density lipoprotein; AST, aspartate aminotransferase; ALT, alanine aminotransferase.

## Data Availability

The data presented in this study are contained within the article.
